# Case Report: A rare phenomenon of venlafaxine induced vocal tics

**DOI:** 10.3389/fpsyt.2026.1797640

**Published:** 2026-03-16

**Authors:** Mouath A. Alturaymi, Kholoud Alnakshabandi

**Affiliations:** 1 Department of Psychiatry, King Saud University Medical City, King Saud University, Riyadh, Saudi Arabia; 2 Department of Psychiatry, College of Medicine, King Saud University, Riyadh, Saudi Arabia

**Keywords:** antidepressants, anxiety, depression, neuropsychiatric, side effects, tics, venlafaxine, vocal

## Abstract

**Background:**

Venlafaxine is a serotonin norepinephrine reuptake inhibitor, an effective medication widely used in many psychiatric conditions, including major depressive disorder and generalized anxiety disorder. It is a relatively safe and tolerated medication. Despite that, it has some side effects affecting many systems, including nausea, insomnia, and tremors. Also, it has some neuropsychiatric side effects, such as akathisia. Venlafaxine induced akathisia has been reported in some studies. Several antidepressants have been associated with some neuropsychiatric manifestations, including tics, usually motor, but not vocal. In our study, we reported a rare side effect of venlafaxine, which is venlafaxine-induced vocal tics.

**Case presentation:**

A 57-year-old women was diagnosed with anxiety and started on venlafaxine with a good response for three months. Then she stopped it. Her anxiety symptoms started to appear again. Then she re-initiated venlafaxine again. A few days afterwards she started to develop vocal tics that are frequent and distressing to the patient. The Yale Global Tic Severity Scale (YGTSS) score at presentation was 22 (Total Tic Score), indicating moderate severity, with an impairment score of 20. Causality assessment using the Naranjo Adverse Drug Reaction Probability Scale yielded a score of 7, indicating a probable relationship between and the vocal tics.

**Discussion:**

To our knowledge, this appears to be the first reported case of venlafaxine induced vocal tics. On the other hand, there were previous reports on Sertraline and Mirtazapine. Previous studies reported associations of tics with SSRIs and atypical antidepressants but not SNRIs. Also, our study is the first study that reported “vocal” tics in association with antidepressant. The proposed mechanism involves a complex interaction between serotonergic augmentation and dopaminergic dysregulation in fronto-striatal circuits, in which increased serotonin levels may indirectly modulate dopamine concentrations to express tics in susceptible individuals.

**Conclusion:**

Our findings suggest that venlafaxine may induce vocal tics. Physicians should consider venlafaxine as a potential cause when patients develop vocal tics while on treatment. Early detection and discontinuation of the medication can prevent complications and lead to symptom resolution.

## 1 Introduction

Venlafaxine is a serotonin–norepinephrine reuptake inhibitor (SNRI) that is a commonly prescribed antidepressant and is considered one of the safest and most effective psychotropic medications (1, 2). venlafaxine has various side effects, most commonly nausea, dizziness, and insomnia. Weight gain and sexual dysfunction are also notable side effects (3). Regarding neuropsychiatric side effects, one report described a patient who developed akathisia after using venlafaxine for depression (4). Moreover, a female patient taking venlafaxine developed significant akathisia and abnormal upper limb movements, which highlights the presence of neuropsychiatric side effects of this medication (5). Somnolence and tremor have also been reported among the notable neuropsychiatric adverse effects (6).

Tics are defined as sudden, quick, and recurrent movements (motor tics). Phonic tics, commonly called vocal tics, are sudden and repetitive sounds that can vary in frequency, intensity, and complexity. They are divided into simple phonic tics (such as sniffing, throat clearing, or grunting) and complex phonic tics, which involve meaningful speech, such as repeating others’ words (echolalia), repeating one’s own words (palilalia), or shouting inappropriate language (coprolalia). While the term “vocal tics” is widely used, “phonic tics” is more accurate, as not all sounds involve the vocal cords; some are produced using the lips, tongue, or teeth, for example, whistling, blowing, or making noises that mimic flatulence (7).

Some antidepressants have been reported to be associated with tics. For example, mirtazapine was prescribed to a 15-year-old boy with depressive and post-traumatic stress symptoms. Two days after initiation, he developed tics in the form of involuntary repetitive movements in the eyebrows and both legs (8). These symptoms disappeared the day after discontinuing mirtazapine (8). Although the exact cause of tics remains unknown and the mechanism behind mirtazapine-associated tics is unclear, it has been proposed that they may be related to increased dopamine levels due to the complex interplay among the serotonin, noradrenaline, and dopamine systems (8).

In addition, another study reported a 16-year-old girl with depression who developed motor tics after starting sertraline, which resolved after discontinuation (9). Similarly, a 35-year-old woman treated with sertraline for major depressive disorder comorbid with obsessive-compulsive disorder developed motor tics that resolved after stopping the medication (10).

Studies exclusively reporting vocal tics induced by antidepressants, specifically, venlafaxine, are limited, which contributes to the novelty of this report. Our study presents a case of venlafaxine-related vocal tics. By reporting this rare phenomenon, we aim to provide clinical insights that may inform both research and future practice.

## 2 Case presentation

A 57-year-old female patient diagnosed with generalized anxiety disorder according to the Diagnostic and Statistical Manual of Mental Disorders, Fifth Edition (DSM-5) (14) was given venlafaxine in a dosage of 75 mg/day, which was continued for two months. She showed considerable clinical improvement. She stopped the drug, and two months later, the patient’s symptoms of anxiety recurred. She was given the same dosage of venlafaxine, 75 mg/day. Within a few days of restarting the drug, the patient developed uncontrollable and repetitive vocal tic behavior, which consisted of throat-clearing and noises similar to the neighing of a horse. These symptoms were embarrassing and distressing. She did not have a history of tics in childhood, and there were no other medical problems.

A diagnostic work-up and investigations were performed to assess the possible underlying causes, including a complete blood count (CBC), renal and hepatic function profiles, electrolyte panel, and thyroid panel. Vitamin B12 was also checked, in light of its association with various movement disorders, including chorea and tremors, that may present with tic-like symptoms (11). Magnetic resonance imaging (MRI) of the brain was also conducted, and all results came back within normal limits. To assess the possibility of other differential diagnoses for adult-onset tics, including autoimmune encephalopathy and Pediatric Autoimmune Neuropsychiatric Disorders Associated with Streptococcal Infections (PANDAS), While PANDAS is predominantly a pediatric condition, rare case reports have described PANDAS-like presentations in young adults (15). However, it is crucial to acknowledge that PANDAS is not a commonly considered differential for adults, and current guidelines do not support its routine investigation in this age group. In our case, the strong temporal association with the initiation of venlafaxine and the absence of other neurological and systemic symptoms ultimately led to the diagnosis of drug-induced tics. further investigations including serological tests may have been considered; however, the temporal association with the initiation of venlafaxine and the absence of other neurological and systemic symptoms led to the diagnosis of drug-induced tics. The Yale Global Tic Severity Scale was used to assess the severity of the tics, and the results yielded a Total Phonic Tic Score of 22, consisting of Frequency 4/5, Intensity 4/5, Number 4/5, Complexity 5/5, and Interference 5/5, and an Impairment Score of 20, resulting in a Global Severity Score of 42.

The temporal relationship between the medication and the onset of symptoms was assessed using the Naranjo Adverse Drug Reaction Probability Scale (**Table 1**). The total score was 7, which classifies the adverse event as a “probable” drug reaction.

**Table 1:** Naranjo adverse drug reaction probability scale.

Question	Score
1. Are there previous conclusive reports on this reaction?	+1 (Yes)
2. Did the adverse event appear after the suspected drug was administered?	+2 (Yes)
3. Did the adverse reaction improve when the drug was discontinued or a specific antagonist was administered?	+1 (Yes)
4. Did the adverse reaction reappear when the drug was readministered?	+2 (Yes)
5. Are there alternative causes (other than the drug) that could on their own have caused the reaction?	+2 (No)
6. Did the reaction reappear when a placebo was given?	0 (Unknown)
7. Was the drug detected in the blood (or other fluids) in concentrations known to be toxic?	0 (No)
8. Was the reaction more severe when the dose was increased, or less severe when the dose was decreased?	0 (Unknown)
9. Did the patient have a similar reaction to the same or similar drugs in any previous exposure?	-1 (No)
10. Was the adverse event confirmed by any objective evidence?	0 (No)
**Total Score**	**7 (Probable)**

**Figure 1:**
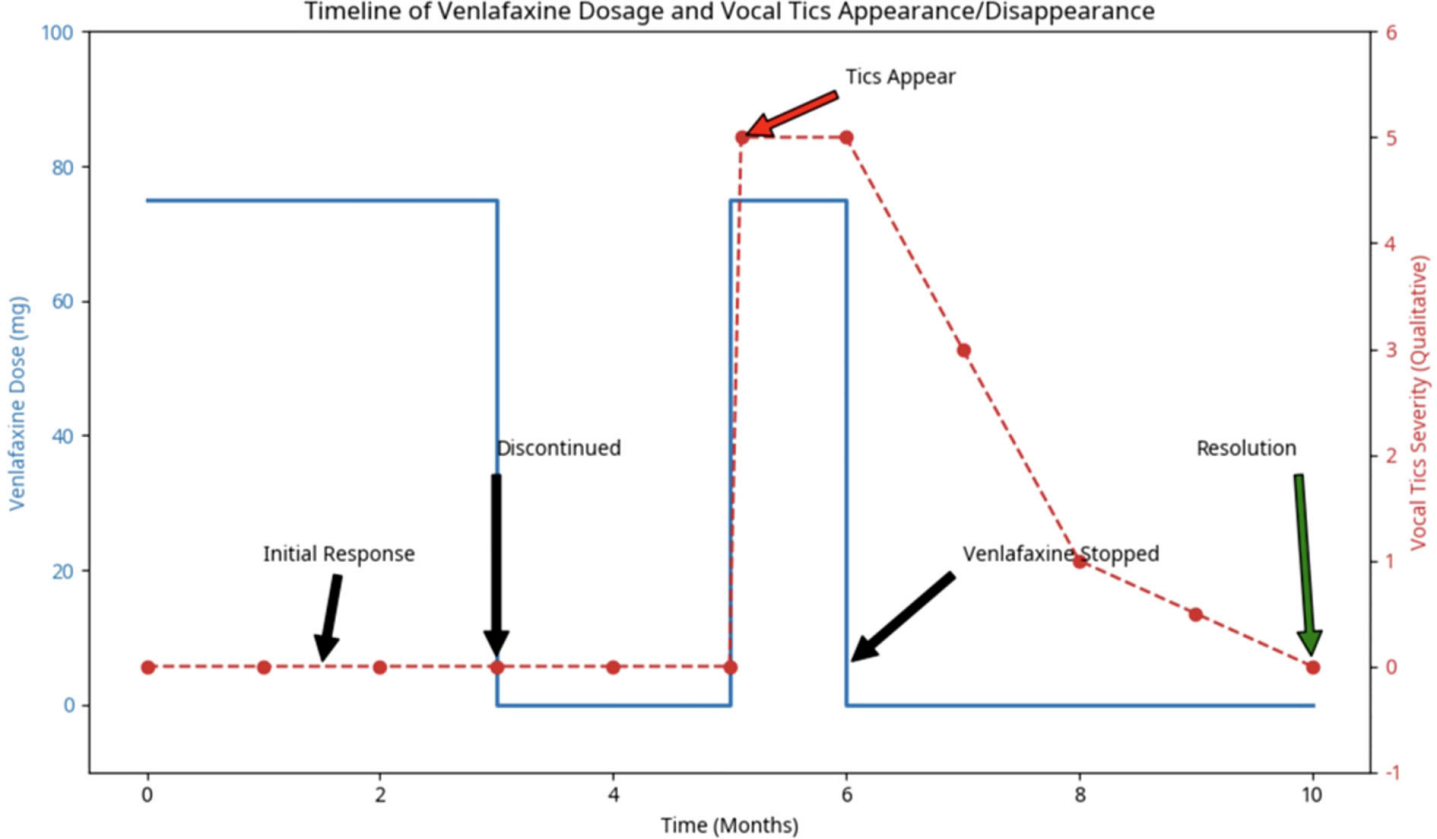
Timeline of venlafaxine dosage and vocal tics appearance/disappearance.

After discontinuing venlafaxine, the patient was placed on an alternative pharmacotherapy regimen consisting of Escitalopram. At the same time, Lorazepam at a dosage of 1 mg once daily for a week was gradually tapered to 0.5 mg once daily for two weeks as needed, in combination with Risperidone 0.5 mg. The patient was followed up over a period of a few months. On subsequent follow-up, the patient continued on a regimen of Escitalopram 10 mg and Risperidone 0.75 mg for a period of six months, showing marked improvement in anxiety symptoms and resolution of vocal tics. The relationship between venlafaxine dosage and the onset and resolution of vocal tics is shown in **Figure 1**. As shown in **Figure 1**, after discontinuation of venlafaxine, there was a decrease in tic symptoms within two months and resolution within four months.

## 3 Discussion

It is most likely that the pathophysiology of antidepressant-induced tics is related to the complex modulation of the dopaminergic systems in the basal ganglia. venlafaxine, by inhibiting serotonin and norepinephrine reuptake, could indirectly affect the dopaminergic systems. For example, increased serotonergic activity could inhibit dopaminergic neurons in the substantia nigra, leading to upregulation or hypersensitivity of postsynaptic dopamine receptors. Disruption of this delicate balance could cause hyperkinetic movements or phonic tics in susceptible people (16–18) The resolution of tics with discontinuation of venlafaxine and the initiation of a low-dose dopamine antagonist (risperidone) supports this mechanism of action.

However, it is also pertinent to state that the simultaneous commencement of low-dose risperidone, a dopamine antagonist, along with the cessation of venlafaxine may be considered a confounding factor in the complete resolution of the patient’s vocal tics and the attribution of the effect solely to the cessation of venlafaxine. Risperidone was initiated to quickly relieve the patient from the disturbing and frequent occurrence of vocal tics and also to address the patient’s anxiety symptoms during the transition from venlafaxine to escitalopram. However, the effect of risperidone on the quick resolution of the patient’s tics cannot be ruled out with complete certainty, which is a limitation of the present case report.

The duration of 2 to 4 months, which is observed in the resolution of vocal tics after discontinuation of venlafaxine, may appear to be prolonged in comparison to the prompt resolution of adverse drug reactions, which is commonly observed. This prolonged duration of time may be attributed to a number of factors, including the complex neurobiological adaptations that result from chronic venlafaxine use, which affects serotonergic and dopaminergic systems, in addition to the restoration of neuronal homeostasis. Furthermore, the pharmacokinetics of venlafaxine, including the active metabolite O-desmethylvenlafaxine, which has an apparent half-life of 10 to 13 hours, indicates that while venlafaxine itself is cleared quickly, the neurochemical sequelae that result, such as desensitization or upregulation of receptors, may require a longer duration to return to normal levels.

A significant point to note in the current case is that the vocal tics appeared after the second dose of venlafaxine, but not after the first dose of venlafaxine. This can be explained on the basis of sensitization/kindling, in which the neurobiological systems have been previously exposed to venlafaxine, making them more susceptible to abnormal regulation after the second exposure. It is likely that after the first exposure to venlafaxine, some subtle subclinical alterations in the regulation of dopamine receptors or in the serotonin-dopamine interaction occurred, which manifested after the second exposure to venlafaxine.

Serotonin-norepinephrine reuptake inhibitors have been suggested as a therapeutic option in the management of anxiety in patients with pre-existing tic disorders, as mentioned by various authors (11). Srour et al. (2008) found that while the use of dopamine-modulating drugs is the first line of management in tic disorders, the management of coexisting anxiety is also important, and in this regard, serotonin-norepinephrine reuptake inhibitors could be used. However, the present case demonstrates a complex and possibly paradoxical response to venlafaxine, which can induce the *de novo* development of vocal tics. It is noteworthy that there have also been suggestions for the use of serotonin and norepinephrine reuptake inhibitors for the treatment of anxiety in patients who have pre-existing tic disorders (12). For instance, Srour et al. (2008) found that although medications that modulate the dopaminergic system are the primary treatment for tics, the treatment of anxiety in such patients is also important and should be included in this framework. However, the case presented here suggests that there may be a complex and paradoxical effect of venlafaxine in some individuals, where there may be a new onset of vocal tics.

The prevention of the adverse effects of the drugs used in the treatment of the patient might require a “start low, go slow” approach. However, in the present case, the adverse effects developed at a standard dosage of the drug (75 mg). Clinicians have to be vigilant about the onset of new involuntary sounds and movements in patients following any alteration in their medication. Though SNRIs are the primary drugs used in the management of anxiety disorders, their use has to be cautious in patients who show any signs of motor and vocal sensitivity.

Some cases have reported tics and other neurological adverse effects related to certain antidepressants, including SSRIs (sertraline) and atypical antidepressants (mirtazapine) (8, 9). On the other hand, our study reveals that an SNRI, venlafaxine, may also be a potential agent causing such adverse effects.

In prior studies, motor tics have been linked to antidepressant use (8). Our study highlights that antidepressants can cause not only motor tics but also vocal tics.

This case report identifies venlafaxine-induced vocal tics, a rare neuropsychiatric phenomenon. Thus, it may serve as the basis for newly established knowledge regarding antidepressant-induced tics and other neurological side effects. Clinicians should keep in mind that both motor and vocal tics may be related to antidepressant use if patients develop such symptoms after initiation of medications.

A strength of our study is that we documented a unique and underrecognized neuropsychiatric adverse effect of venlafaxine, enriching the limited literature in this field. The clear association between medication initiation, onset of vocal tics, and symptom resolution after discontinuation increases the possibility of causality. Furthermore, the use of standardized scales (Naranjo and YGTSS) provides objective evidence for the severity and causality of the adverse reaction, aligning with the CARE guidelines for clinical case reporting (13).

However, case report studies cannot ensure the generalization. Being a single case, this report cannot definitely establish causality between venlafaxine and vocal tics. Therefore, further studies with stronger study designs are recommended to confirm this association.

## 4 Conclusion

This case reveals a rare yet clinically significant adverse effect of venlafaxine. These findings suggest that venlafaxine should be considered as a possible etiologic agent in patients who present with sudden involuntary vocalizations. Early recognition and discontinuation of the drug are critical in the management of the condition and resolution of the symptoms.

## Data Availability

The original contributions presented in the study are included in the article/**Supplementary Material**. Further inquiries can be directed to the corresponding author.
